# An Address Delivered at the Birmingham Royal School of Medicine and Surgery

**Published:** 1839-04

**Authors:** 


### Art. XV.

*An Address delivered at the Birmingham Royal School of Medicine and Surgery, at the Third Anniversary Meeting, August* 29, 1838. By Vaughan Thomas, b.d., formerly Fellow and Tutor of Corpus Christi College, Oxford.—*Birmingham*, 1838. 8vo. pp. 61.

This address is in many respects deserving of attention. The learned and reverend author is one of those members of the church in whose varied acquirements, classical, theological, and medical, may be recognized traces of the ancient connexion existing between these studies; and who, by watchful superintendence and unwearied charity, become the most effective allies of medical men in their exertions for the general benefit of the sick and distressed. As the friend and distributor, in many instances, of the bounty of the Rev. Samuel Warneford, he has acquired an intimate practical knowledge of the working of the various medical charities which that most philanthropic gentleman has assisted, or supported, or established, or enriched.* We introduce the name of Dr. Warneford on this occasion not only because he presents an instance of almost unexampled generosity to numerous medical charities, but because, not content with supplying them with the means of doing good, he devotes a large portion of his time, at an age which many would consider as affording an excuse for declining all active labours of such a kind, to inspecting the best-conducted hospitals, and to assisting in the management of several of the institutions to which he has secured prosperity; where, by the example he gives of unbounded liberality, conjoined with the most exact and scrupulous economy, he contributes to establish such a system of government as may secure their extensive and permanent utility. Wisely deeming, also, that no more important

* To the Radcliffe Lunatic Asylum, near Oxford, Dr. Warneford and his sister have contributed not less than fifteen thousand pounds! The Leamington Hospital has also received three or four thousand pounds from the same source; and we believe that even these large donations form but items among the acts of benevolence exhibited by this distinguished family towards the public institutions of Oxfordshire, Warwickshire, and Gloucestershire.

benefit can be conferred on medical charities than supplying them with carefully educated medical officers, Dr. Warneford has directed some portion of the wealth which he administers to so many good purposes, to encouraging medical education. His presents of books to the library of the Birmingham Medical School have been numerous; and his donation of £1000 to the same school is the immediate occasion of the address before us, the object of which is to explain the conditions of the grant. It is to be applied for ever to affording two annual prizes for the best Essay on Anatomy, general, descriptive, and pathological, or on Physiology, written "in a practical or professional manner, and according to those evidences of facts and phenomena which anatomy, physiology, and pathology so abundantly supply; but always and especially with a view to exemplify or set forth by instance or example the wisdom, power, and goodness of God, *as revealed and declared in Holy Writ.*"

On this text Mr. Vaughan Thomas discourses eloquently, learnedly, and fully; with many scholastic divisions, and not a few unusual terms. The address is printed with marginal capitals, the leaders, each, of five lines of type: and marginal headings are let in after a somewhat antiquated fashion. With all this, the address is less correctly printed than such an imposing appearance should ensure; and even a table of eight flagrant *errata* does not comprehend more than half of the typographical blunders. We should not notice this but for the curious peculiarity of the pages, the aspect of which probably reflects in miniature some venerable and seldom turned folios in the Bodleian, for which Mr. Thomas has a scholar's affection. Of Dr. Warneford's truly pious intentions, and of Mr. Vaughan Thomas's earnest and able exposition of them, it would ill become us to speak in any terms but those of sincere respect. How far medical students in a provincial school may be able to comply with the peculiar conditions, admits of question; and we confess that we are so doubtful of the benefit derived by religion from the constant practice of writers, now-a-days, to quote every circumstance in nature as proving the precise attributes of the Deity, as to fear the subject of revelation will suffer some damage if the same violence be done it after the same fashion. The zeal and warmth with which every function of every plant and animal is now laid hold of as a proof of the wisdom and power of the Creator, after the manner of the Bridgwater Treatises, implies an accusation levelled against some presumed mass of unbelief on such points: and we are sometimes disposed to exclaim, "quis vituperavit?" These instances are often ill selected; sometimes absurd. Still more difficult is it, even for the most learned divine and accomplished naturalist, to select with judgment appropriate analogies (the existence of which we are far from denying) between the will of God as revealed in the works of nature and in Holy Writ; and the committal of such a task to a student is a somewhat delicate matter, and will require the utmost circumspection in the teachers and governors of the school. We do not doubt, however, that this will be duly considered and well attended to in the Birmingham school; to the respectability, efficiency, and prosperity of which we have had other occasions of bearing testimony.

The *curriculum* proposed in the University of London has, we believe, led to the adoption of some alterations in most of the provincial schools; and the general result will unquestionably be that the literary attainments of the medical pupils and medical men will be increased. We do not mean that scholars will more abound among them ; but that fewer of them will be imperfectly prepared for entering on the studies of medicine and surgery. It would be wrong to conceal that medical practitioners in the country view the provincial schools with some dislike, and avow their belief that they tend, by rendering medical education too cheap and easy, to fill country towns with ill-educated practitioners. This, if true, would constitute a very serious objection to them; but it is very difficult to establish either the correctness or incorrectness of the allegation, and we are ourselves disposed to lay very little stress upon it. We have some fears that the discipline of the country schools is inefficient; but even in this respect they cannot exceed some of the schools of London, where the pupils constitute a democracy, to which, for the meanest purposes, the professors having once willingly bowed must be slaves for ever In all schools of medicine, we apprehend, there is some little tendency in the teachers to anticipate the period when the pupils will be practitioners, and seek the assistance of their honoured preceptors; and to this kind of influence, from local circumstances, a country teacher is peculiarly exposed. The check and remedy, however, lie in the hands of the examining bodies of the profession ; and we see no room to doubt their vigilance.

We perceive, by the last Annual Report of the Birmingham School, that the nobility and gentry of the neighbourhood take an interest in its progress; assist it by donations; and encourage its active friends by attending the periodical meetings. The school has thus been enabled to form museums of anatomy and natural history, and to offer many helps to the pupils, of which, before the establishment of University College, London, they had not a free command in any school in the metropolis.

It is with some unwillingness that we allude to the portion of the address in which, in a pardonable zeal for the welfare of King's College, London, some of the teachers of anatomy and physiology, in other schools, are accused, by " mingling lessons of infidelity with anatomical and physiological instruction,"—of doing what they can " to rob God of his glory, and man of his gratitude; moral virtue of its best support; human reason of its surest guidance; and professional attainments of their brightest honour." This charge was made, it appears, in the presence of many medical men; and in our opinion it ought to have been noticed at the time. We conceive it to be quite unfounded. Medical teachers, doubtless, differ, no less than other teachers, respecting many religious doctrines and some articles of belief; but the general accusation of infidelity, and of mixing up such infidelity with their professional lectures, is too serious to be made without adducing the particular instances, which we think it would not be easy to do.

